# Mining Feature of Data Fusion in the Classification of Beer Flavor Information Using E-Tongue and E-Nose

**DOI:** 10.3390/s17071656

**Published:** 2017-07-19

**Authors:** Hong Men, Yan Shi, Songlin Fu, Yanan Jiao, Yu Qiao, Jingjing Liu

**Affiliations:** College of Automation Engineering, Northeast Electric Power University, Jilin 132012, China; menhong@neepu.edu.cn (H.M.); 2201500430@neepu.edu.cn (Y.S.); 2201500474@neepu.edu.cn (S.F.); 2201600437@neepu.edu.cn (Y.J.); 2201600453@neepu.edu.cn (Y.Q.)

**Keywords:** e-tongue, e-nose, data fusion, feature mining, variable accumulation, beer

## Abstract

Multi-sensor data fusion can provide more comprehensive and more accurate analysis results. However, it also brings some redundant information, which is an important issue with respect to finding a feature-mining method for intuitive and efficient analysis. This paper demonstrates a feature-mining method based on variable accumulation to find the best expression form and variables’ behavior affecting beer flavor. First, e-tongue and e-nose were used to gather the taste and olfactory information of beer, respectively. Second, principal component analysis (PCA), genetic algorithm-partial least squares (GA-PLS), and variable importance of projection (VIP) scores were applied to select feature variables of the original fusion set. Finally, the classification models based on support vector machine (SVM), random forests (RF), and extreme learning machine (ELM) were established to evaluate the efficiency of the feature-mining method. The result shows that the feature-mining method based on variable accumulation obtains the main feature affecting beer flavor information, and the best classification performance for the SVM, RF, and ELM models with 96.67%, 94.44%, and 98.33% prediction accuracy, respectively.

## 1. Introduction

The consumption of beer, as a beverage, ranks third in the world after water and tea. It is rich in various amino acids, vitamins, and other nutrients needed by the human body [1,2], which is euphemistically known as ‘liquid bread’. Barley germination is the main raw material for beer brewing, which makes beer a low-alcohol and high-nutrition drink. Additionally, it promotes digestion, spleen activity, appetite, and other functions [3,4,5].

Flavor information is one of the reference factors that reflects the beer’s features, which consists of taste and olfactory information. Due to different manufacturing processes, both the taste and the smell of different beers are different. According to consumer preference, they choose beers with different flavors. Therefore, accurately and efficiently identifying different beers, and finding important features, are particularly significant. Meanwhile, it is also meaningful for quality control, storage, and authenticity recognition. A crucial observation was obtained in the psychology literature that the intensity of the senses could be overlapped, and people usually mistake volatile substances as ‘taste’ [6]. When we cannot smell, it is difficult to distinguish apple and potato, red wine and coffee. The food odor can stimulate people to salivate, which improves our sensation. When drinking fruit juice with the nose squeezed, sweet and sour can be felt by the tongue. Setting the nose free after drinking, the fruit juice flavor information will appear, so the sensory experience of food must be fully dependent on both the tongue and nose [7]. The conventional physical and chemical analysis methods cannot reflect the flavor characteristics of beer [8,9,10]. The most used method is sensory evaluation [11], but this method is quite subjective since the evaluation result changes with the physical condition and environment. It is time-consuming and has low efficiency. As objective and effective intelligent bionic instruments that are easy to operate, offer high precision, and are time-saving, among other advantages, e-tongue [12,13,14] and e-nose [15,16,17] are gradually replacing the traditional detection methods. 

The e-tongue and e-nose could be applied to analyze the beer [18,19]. However, the flavor features of beer are complicated due to its composition and concentration. Therefore, the e-tongue and e-nose fusion system has a significant advantage of obtaining comprehensive information of taste and olfactory characteristics. The combined information based on the instruments is called data fusion [20]. The data-level (low-level) fusion combines the original sensing information of multiple detection instruments to obtain new data. The feature-level (medium-level) fusion combines features extracted from the original sensing information of multiple detection instruments. The decision-level (high-level) fusion combines sensor information after each sensor has made a preliminary determination, then fuses that information to obtain a final decision. Multi-sensor data fusion based on e-tongue and e-nose has been used widely, for instance, in the blending ratio prediction of old frying oil [21], classification of different honey and rice samples [22,23], nondestructive detection of fish freshness [24], and the evaluation of tea and strawberry juice flavor [25,26]. The previous studies showed that multi-sensor data fusion in the classification of food and quality assessment were much closer to the human perception mode and improved the analysis results. However, it also brings some irrelevant information, and even noisy information. The studies cannot give an effective feature selection method, which could lead to an unfavorable final classification and prediction, and increase the complexity of the model prediction. More importantly, we should adopt fewer features to reduce the sample detection difficulty and detection time, and the best fusion and identification methods are applied to improve the detection efficiency and classification accuracy rate of samples in real projects. For the feature method research, experts took advantage of GA-PLS to reduce the number of variables of electrochemical signals [27], extracted the features from sensor response signals in the time and frequency domains [28], optimized the number of channel inputs of the olfactory nervous system bionic model based on PCA [29], selected the variable characteristics of the multi-sensor based on the analysis of variance (ANOVA) [30], and applied multidimensional projection techniques (interactive document map) to analyze the capacitance data [31]. These studies were significant for reducing the feature dimension and achieving high-precision prediction of the sample. However, studies lacked a method to assess the importance of variables, and little information on the variables’ behavior and correlation were offered.

**Figure 1:**
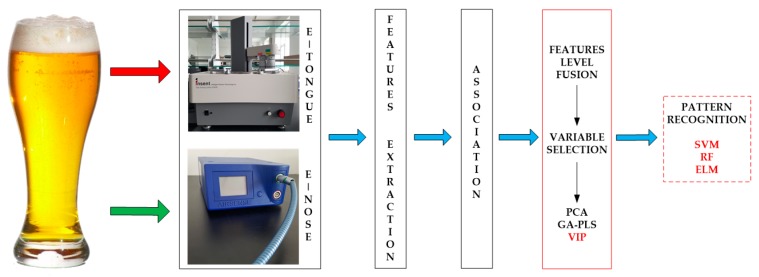
The graphical abstract for this paper.

This study provides a feature-mining method to obtain the best form of expression affecting beer flavor information. It improves the accuracy rate of identification and reduces the complexity of model prediction. Under the practical application background, it is significant that models are fast and accurate. Here, we select the SVM, RF, and ELM as the appraisal models, which have good generalizability and fast running time. We observe the classification performance of evaluation models to find the main feature and the variable’s behavior, and contrast the analysis results of the different evaluation models to verify the validity and universality of the method. Figure 1 shows the technical route for this paper.

## 2. Materials and Methods 

### 2.1. Beer

Five different beers were used in this study, and their alcohol degree, original wort concentration, and raw materials were obtained from the beer bottle labels. Table 1 lists all of these.

### 2.2. Data Acquisition of Intelligent Bionic Detection

#### 2.2.1. E-Tongue Data Acquisition

**Figure 2:**
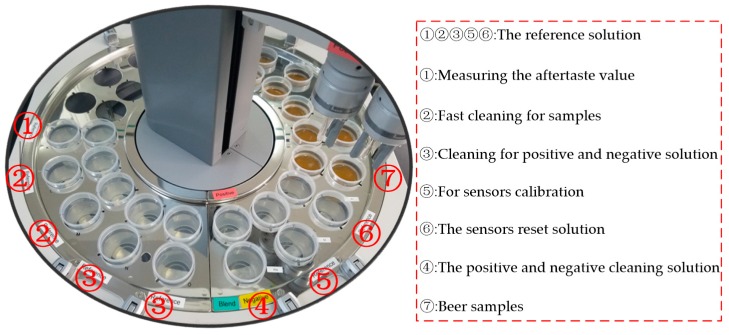
The SA-402B e-tongue system.

The SA-402B e-tongue, developed by the Japan Insent Company, was used to gather beer taste information. The instrument includes a sensor array, an automatic detection system, a data acquisition system, and data analysis software. The sensor array consists of five taste sensors, and each sensor is composed of a unique artificial lipid-based membrane. Two Ag/AgCl electrodes containing an inner solution containing 3.33 M KCl and saturated AgCl were used for the reference electrode. The sensor AAE was applied to detect umami substances. The sensor CT0 was applied to detect salty substances. The sensor CA0 was applied to detect sour substances. The sensor C00 was applied to detect bitter substances. The sensor AE1 was applied to detect astringent substances. The positive sensor array consisted of C00, AE1, and a reference electrode. The negative sensor array consisted of CT0, CA0, AAE, and a reference electrode. Figure 2 shows the SA-402B e-tongue system.

The sample solution, reference solution, positive cleaning solution, and negative cleaning solution were put into the reagent tank. The automatic detection device manipulated the robot arm to collect the sample’s taste information by setting the system parameter. When the taste substances were absorbed by the unique artificial lipid-based membrane, the potential difference between the working electrode and the reference electrode was measured. Forty milliliter beer samples were placed into the clean measuring cups. Before the test began, the sensor was cleaned in the positive and negative cleaning solution for 90 s, after which it was cleaned in the reference solution for 120 s, and then repeated in another reference solution. After the balance was reached in the reference solution, the test was started. The sensor tested each sample for 30 s, then the sensor was cleaned sensor twice, quickly, and returned to the reference solution to measure the aftertaste value (cpa), the measurement was completed once. After each measurement, the sensors were cleaned automatically. Six samples of each beer were prepared for measuring three times. Finally, a total of 90 samples were obtained. The experimental temperature was 20 ± 0.5 °C, and the relative humidity was 65 ± 2% RH. The intensity value of each sensor at the 30th second was extracted and analyzed in this study.

#### 2.2.2. E-Nose Data Acquisition

**Figure 3:**
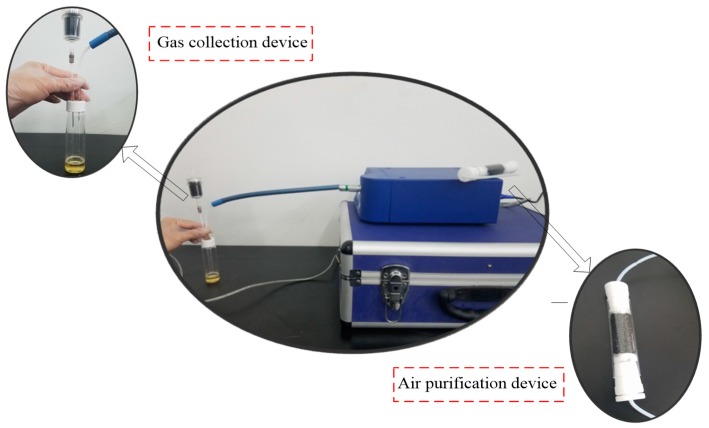
The PEN3 e-nose system.

The PEN3 e-nose, developed by the Airsense Analytics Inc. (Schwerin, Germany), was used to gather beer olfactory information. The instrument includes a gas collection device, a gas detection unit, and an air purification device. The gas detection unit includes a sensor array, and a pattern recognition analysis and processing system. The sensor array contains 10 metal oxide gas sensors, which can achieve the detection of olfactory cross-sensitive information. The components to be detected by sensors were listed as below: aromatic (W1C), hydrocarbon (W5S), aromatic (W3C), hydrogen (W6S), arom-aliph (W5C), broad-methane (W1S), sulfur-organic (W1W), broad-alcohol (W2S), sulfur-chlorine (W2W), and methane-aliphatic (W3S). Figure 3 shows the PEN3 e-nose system.

Five milliliters of a beer sample was put into a cork-tightened 50-mL sampling chamber for 10 min to ensure sufficient volatility. Before the test began, the gas chamber was cleaned with a gas flow to normalize the sensor signal, which was filtered by active charcoal at a flow rate of 300 mL/min for 60 s. The detection time was 80 s at the gas flow speed of 300 mL/min, so that the sensor reached a stable value. The sensor response value was defined as G/G0 (G0/G), where G is the conductivity of the sensor when the sample to be tested entered the sensor gas detection unit and G0 is the conductivity of the sensor when the pure gas entered the sensor gas detection unit. Eighteen samples of each beer were prepared for measurement. Finally, a total of 90 samples were obtained. The experimental temperature was 20 ± 0.5 °C, and the relative humidity was 65 ± 2% RH. The intensity value of each sensor at the 60th second was extracted and analyzed in this study. 

### 2.3. Variable Selection

Principal component analysis (PCA) is a multivariate statistical analysis method which can transform the data into a new coordinate system, converting the multivariate information into several synthetic variables [32]. PCA preserves the useful information of the original variable, reducing the dimension of the multidimensional dataset, and extracts the principal component. The number of principal components is calculated according to the maximum variance principle. We determined the number of principal components according to the cumulative contribution rate and practical requirements. In this work, in order to obtain as much information as possible from the original fusion set, we made sure that the principal component with a cumulative variance contribution was 99%.

The feature variables can be screened by genetic algorithm-partial least squares (GA-PLS) to remove redundant variables for constructing the classification model [33,34]. In the process of variable selection, a randomization test is used to determine whether it can be applied; usually, the randomization test value is less than 5. As the number of variables increases, the cross-validated exceptions variance (CV%) value gradually increases to reach a maximum, and finally maintains a relatively stable state. Meanwhile, the root mean square error of cross-validation (RMSECV) gradually decreases to reach a minimum, and finally maintains a relatively stable state. In the calculation process, the chromosome corresponds to the highest CV%, and smallest RMSECV is the best optimal variable subset. 

In the PLS, the explanatory ability of the independent variable to the dependent variable is measured by the variable importance of projection (VIP) scores [35]. The marginal contribution of the independent variable to the principal component is called VIP. The VIP definition is based on the fact that the explanatory ability of the independent variable to the dependent variable is passed through t, and if the explanatory ability of t to the dependent variable is strong, and the independent variable plays a very important role for t, we think that the explanatory ability of the independent variable to the dependent variable will be large. In this study, the variable importance of the e-tongue and e-nose fusion set is sorted based on the VIP scores. 

### 2.4. Multivariate Analysis

#### 2.4.1. Support Vector Machines (SVM)

SVM was first proposed by Cortes and Vapnik [36], and is a supervised learning model for classification and regression. The main idea is to establish a classification hyperplane as a decision plane. The SVM uses the kernel function to map the data to the high-dimensional space, making it as linear as possible. The kernel functions include linear kernel, polynomial kernel, radial basis kernel (RBF), Fourier kernel, spine kernel, and sigmoid nucleus in SVM. Compared with the kernel function and previous studies, the RBF kernel function gave an excellent classification performance [37,38,39]. Whether the sample is small or large, high dimension or low dimension, the RBF kernel function is applicable. Therefore, this paper used the RBF as the SVM classification kernel function. 

The SVM algorithm proceeds as follows:

Set the detected data vector to be N-dimensional, and then the L sets can be represented as $(x_{1},y_{1}),\cdots ,(x_{l},y_{l})\in R^{n}$.

The hyperplane constructed as:

(1) f(x)=ϖ·φ(x)+b

where ϖ is the weight coefficient of the decision plane, φ(x) is a nonlinear mapping function, and b is the domain value for the category division. In order to minimize the structural risk, the optimal classification plane satisfies the condition as:

(2) $$y_{i}\left(\varpi \cdot \varphi (x_{i})+b\right)\geq 1$$

Introducing the nonnegative slack variable $\xi_{i}$, the classification error is allowed within a certain range. Therefore, the optimization problem is translated into:

(3) $$\left\{ \begin{array}{l} \min\frac{1}{2}{\left\|\varpi \right\|}^{2}+c\sum_{i=1}^{n}\xi_{i},c\geq 0\\ \begin{array}{cc} s.t. & y_{i}[(\varpi \cdot \varphi (x_{i})+b)]\geq 1-\xi_{i}, \end{array}\xi_{i}\geq 0 \end{array}\right.$$

where c is the penalty factor to control the complexity and approximation error of the model, and determine the generalizability of the SVM. Here, introducing the Lagrange multiplier algorithm, the optimization problem is transformed into dual form:

(4) $$\left\{ \begin{array}{l} \min\frac{1}{2}\sum_{i=1}^{n}\sum_{j=1}^{n}y_{i}y_{j}a_{i}a_{j}K(x_{i},x_{j})-\sum_{i=1}^{n}a_{i}\\ \begin{array}{cc} s.t. & \sum_{i=1}^{n}y_{i}a_{i}=0,0\leq a_{i}\leq c \end{array} \end{array}\right.$$

where:

(5) $$K(x_{i},x_{j})=(\varphi (x_{i})\cdot \varphi (x_{j}))$$

In this paper, the RBF kernel function is introduced:

(6) $$K(x_{i},x_{j})=\exp{\left(-g\left\|x_{i}-x_{j}\right\|\right)}^{2}$$

where g is the kernel function parameter, which is related to the input space range or width. The larger the sample input space range is, the larger the value is. In contrast, the smaller the sample input space range is, the smaller the value is. The above optimization problem is translated into:

(7) $$\left\{ \begin{array}{l} \min\frac{1}{2}\sum_{i=1}^{n}\sum_{j=1}^{n}y_{i}y_{j}a_{i}a_{j}\exp{\left(-g\left\|x_{i}-x_{j}\right\|\right)}^{2}-\sum_{i=1}^{n}a_{i}\\ \begin{array}{cc} s.t. & \sum_{i=1}^{n}y_{i}a_{i}=0,0\leq a_{i}\leq c \end{array} \end{array}\right.$$

Therefore, the minimization problem depends on the parameters c and g, and the correct and effective selection of parameters would show a good classification performance for SVM. Thus, GA was combined with SVM to optimize the penalty factor c and the kernel function parameter g. The parameters of the GA are initialized as follows: maximum generation was 100, population was 20, the search range of c was 0 to 100, and that of g was 0 to 1000.

#### 2.4.2. Random Forests (RF)

RF is a nonlinear classification and regression algorithm, which was first proposed by Tin Kam in 1995. In 2001, Breiman conducted a deeper research [40]. The method combines bootstrap aggregating and random subspace successfully. The essence of RF is a classifier that contains a number of decision trees that are not associated with each other. When the data is input into a random forest, the classification result is recorded by each decision tree. Finally, the category of data is voted by decision trees. The RF shows good efficiency in practical applications, such as image processing, environmental monitoring, and medical diagnosis [41,42,43].

The RF algorithm proceeds as follows:

(1) Using bootstrap sampling to generate T training sets $S_{1},S_{2},\cdots ,S_{T}$ randomly;
(2) Each training set is used to generate the decision tree $C_{1},C_{2},\cdots ,C_{T}$. The value of the split property set for each tree is mtry. The mtry value is the square root of the number of input variables. In general, the value of mtry remains stable throughout the forest development process;
(3) Each tree has a complete development without taking pruning;
(4) For testing set X, each decision tree is used to test and obtain the category $C_{1}(X),C_{2}(X),\cdots ,C_{T}(X)$; and
(5) The category of the testing set is voted by decision trees.

#### 2.4.3. Extreme Learning Machine (ELM)

Extreme learning machine (ELM) is a new algorithm for regression and classification, which was first proposed by Huang of Nanyang Technological University [40]. The essence of ELM is a single hidden layer feed-forward neural network (SLFN). The difference with other SLFN is that ELM randomly generates the connection weights (w) and threshold (b), and without adjustment in the training process. The optimal results can be obtained by adjusting the number of neurons in the hidden layer. Compared with the traditional training methods, this method has the advantages of fast learning speed and good generalization performance. In recent years, ELM has gained attention widely, such as with on-line fault monitoring, price forecasting, and control chart pattern recognition [44,45,46].

The ELM algorithm proceeds as follows:

N samples are described by $\left(x_{i},t_{i}\right)$, where $x_{i}={\left[x_{x1},x_{x2},\cdots ,x_{xn}\right]}^{T}\in R^{n}$, $t_{i}={\left[t_{i1},x_{i2},\cdots ,t_{im}\right]}^{T}\in R^{m}$. The activation function of neurons in the hidden layer is G. The ELM model can be represented as:

(8) $$f_{\tilde{N}}=\sum_{i=1}^{\tilde{N}}\beta_{i}G(a_{i},b_{i},x_{j})=t_{j},j=1,\cdots ,N$$

where $a_{i}={\left[a_{1},a_{2},\cdots ,a_{n}\right]}^{T}$ is the weight vector of the ith hidden layer node and the input node. $\beta_{i}={\left[\beta_{1},\beta_{2},\cdots ,\beta_{n}\right]}^{T}$ is the weight vector of the ith hidden layer node and the input node. $b_{i}$ is the threshold of the ith hidden node. $\tilde{N}$ is the number of hidden neurons. Equation (8) can be abbreviated as:

(9) Hβ=T

where:

(10) $$H(a_{1},\cdots ,a_{\tilde{N}},b_{1},\cdots ,b_{\tilde{N}},x_{1},\cdots ,x_{N})={\left[\begin{array}{ccc} G(a_{1},b_{1},x_{1}) & \cdots & G(a_{\tilde{N}},b_{\tilde{N}},x_{1})\\ \vdots & \vdots & \vdots \\ G(a_{1},b_{1},x_{N}) & \cdots & G(a_{\tilde{N}},b_{\tilde{N}},x_{N}) \end{array}\right]}_{N\cdot \tilde{N}}$$

(11) $$\{\begin{array}{c} \beta ={\left[\beta_{1}^{T}\cdots \beta_{\tilde{N}}^{T}\right]}_{\tilde{N}\cdot m}\\ T={\left[t_{1}^{T}\cdots t_{N}^{T}\right]}_{N\cdot m} \end{array}$$

where H is the output matrix of the hidden layer, and the output weights can be obtained by solving the least squares solution of the linear equations:

(12) $$\left\|H\beta -T\right\|=\left\|HH^{+}T-T\right\|=\underset{\beta }{\min}\left\|H\beta -T\right\|$$

The least squares solution as:

(13) $$\beta =H^{+}T$$

where $H^{+}$ is the Moore-Penrose generalized inverse of the hidden layer output matrix H.

### 2.5. Allocation of Datasets and the Model Prediction Process

The 90 samples of original data were divided into two groups randomly. One group included 72 samples, which was used as a training set to build the model. The remaining 18 samples were used as a testing set to verify the classification performance of the model.

In SVM, in order to avoid over-learning and insufficient learning when searching the best parameters, the fitness function value of the GA was the highest accuracy rate of the training set under five-fold cross-validation, and the best c and g were selected when the highest CVAccuracy was obtained. In order to eliminate the impact of randomness, 10 prediction models were established, and then the average of their accuracy rates was used to describe the classification performance of the SVM.

In RF, the mtry value is the square root of the number of input variables. Therefore, the mtry value of single e-tongue, single e-nose, the dimensionality reduction set by PCA, and the feature screening set by GA-PLS were 3. The mtry value of original fusion set was 4. The 20 subsets based on variable accumulation, the mtry values of 1–3, 4–8, 9–15, and 16–20 were 1, 2, 3, and 4, respectively. Then, we observed the classification performance of RF by changing the number of random forest decision trees. The number of decision trees was taken from 2–100 at intervals of 2. In order to eliminate the impact of randomness, 10 prediction models were established, and then the average of their accuracy rates was used to describe the classification performance of the RF under the current decision tree.

In ELM, when the activation function of neurons in the hidden layer was determined, we changed the number of hidden layer neurons to observe the ELM classification performance. The number of hidden layer neurons was taken from 2–100 at intervals of 2. In order to eliminate the impact of randomness, 10 prediction models were established, and then the average of the accuracy rates was used to describe the classification performance of the ELM under the current neurons.

## 3. Results and Discussion

### 3.1. Pre-Processing

**Figure 4:**
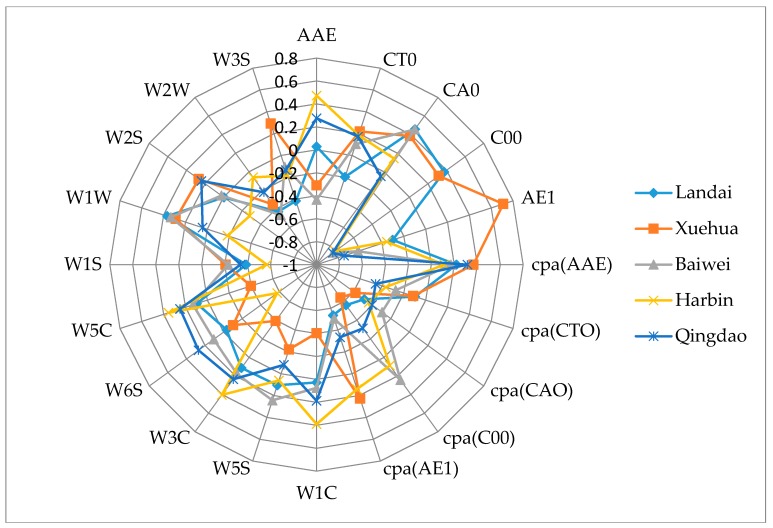
A radar plot of the different sensors to five beer samples.

The detection data of the e-tongue and the e-nose contained 10-dimension feature variables, respectively. Data from the two systems were combined to form new data for describing the beer flavor information. A normalization between (−1, +1) was implemented on the original feature set from the different sensors of the e-tongue and e-nose. Figure 4 shows the averaged-value radar plot of the normalized values. According to the sensor response information, it was difficult to identify the different samples, and the relationship for each sensor was extremely complex. It was difficult to find the main features and their contribution to the beer flavor information. Thus, mining the data features and obtaining the variables’ behavior are particularly important for distinguishing beer brands correctly.

### 3.2. Extraction of Sensor Feature Variables

In order to acquire as much information as possible from the original fusion set, the 10 principal components were extracted by PCA, and the accumulated variance contribution rate was as high as 99.99%. 

**Figure 5:**
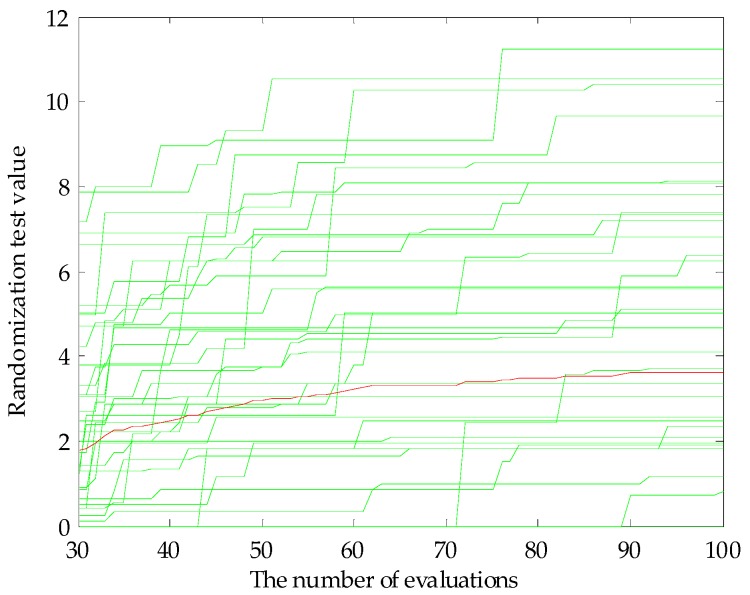
Randomization test result.

**Figure 6:**
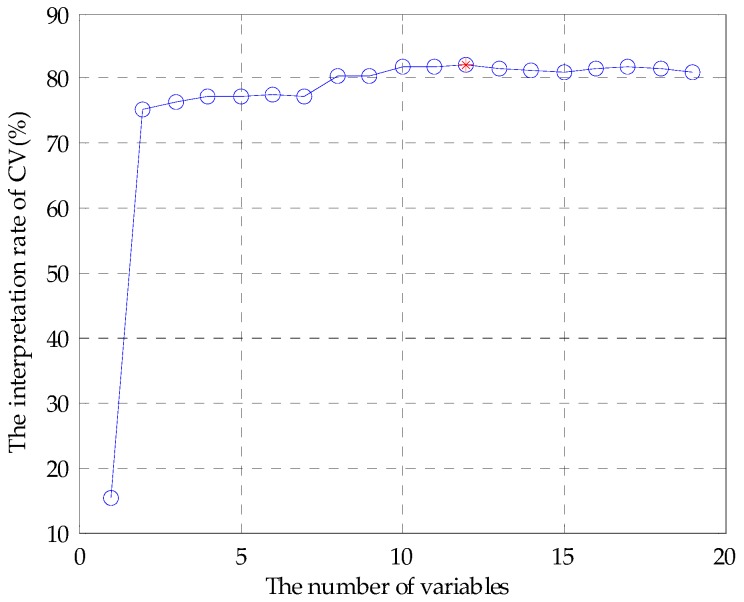
CV% change curve.

**Figure 7:**
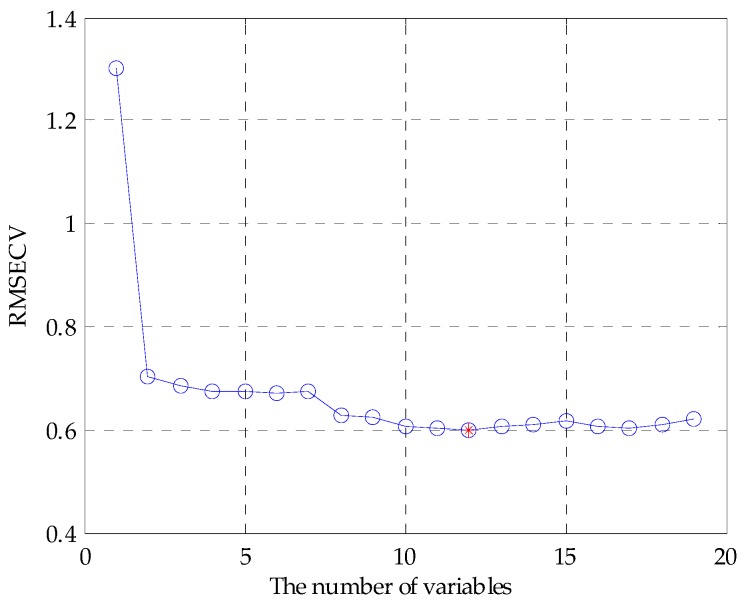
RMSECV change curve.

Before applying the GA-PLS to select variables, a randomization test was required to determine whether it could be applied. Figure 5 shows that randomization test result of the original fusion set. It can be seen that the randomization test value was less than 5, indicating that the GA-PLS was reliable. Figure 6 shows the GA-PLS search process for the best number of variables, it can be seen that the CV% increased rapidly and then gradually slowed down as the number of variables increases. When CV% reached a maximum 82.169%, the number of variables reached 12, and the number of variables continued to increase, the CV% decreased slightly and then stayed in a relatively stable state. On the contrary, in Figure 7, RMSECV decreased rapidly, and then slowly with the increase in the number of variables. When the number of variables reached 12, the RMSECV arrived at its minimum value of 0.5937, and then as the number of variables continued to increase, RMSECV increased slightly and maintained a relatively stable state. Finally, 12 feature variables were extracted from the original fusion set, which were CA0, C00, AE1, AAE, cpa(C00), W1C, W5S, W6S, W1S, W1W, W2S, and W2W.

**Figure 8:**
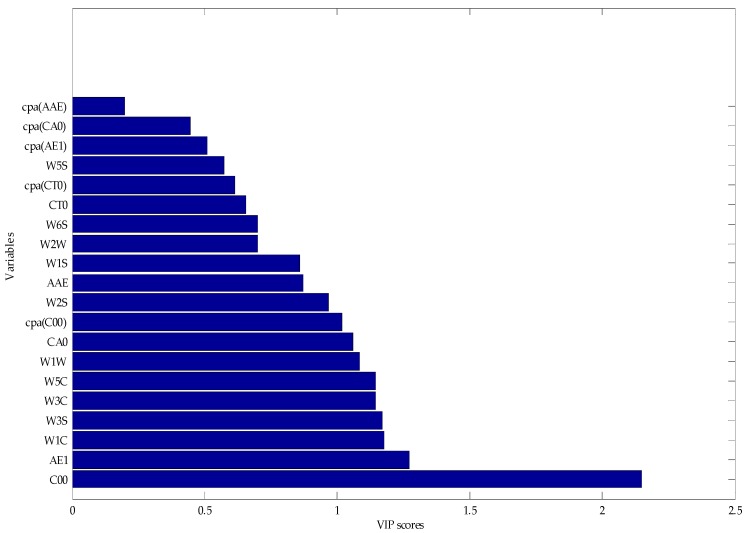
Ranking the importance of variables based on the VIP score.

Figure 8 shows the VIP score of the original variables of the e-tongue and e-nose. C00 and AE1 had larger VIP scores than the other variables, and this means that bitter and astringent were significant for beer classification. Cpa(AAE) and cpa(CA0) had smaller VIP scores, and this means that the aftertaste of umami and sour were less important for beer flavor. Thus, we generated the variable subsets which could be used to build the classification models. Each subset was generated with those variables based on the best VIP score. Subset #1 included C00, subset #2 included C00 and AE1, and subset #20 contained all the variables of the e-tongue and the e-nose. We then gradually accumulated the number of variables, and observed the classification results of the models to achieve the purpose of filtering redundant information [47]. 

### 3.3. Results of the Models

**Figure 9:**
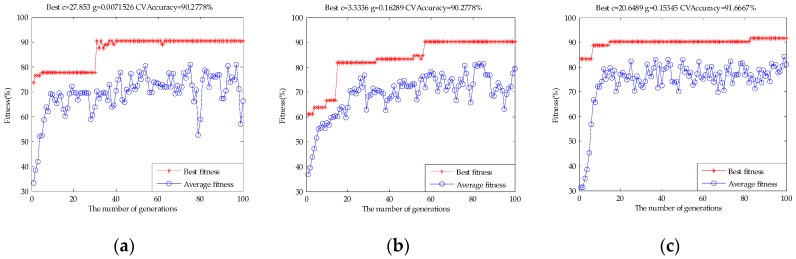
The parameter optimization fitness curve of the SVM: (**a**) e-tongue; (**b**) e-nose; and (**c**) the original fusion set.

**Figure 10:**
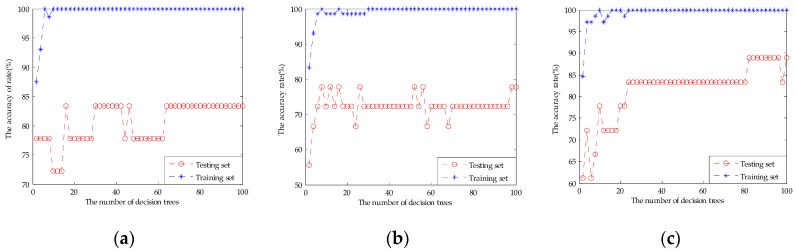
The classification performance of the RF based on the number of decision trees: (**a**) e-tongue; (**b**) e-nose; and (**c**) the original fusion set.

**Figure 11:**
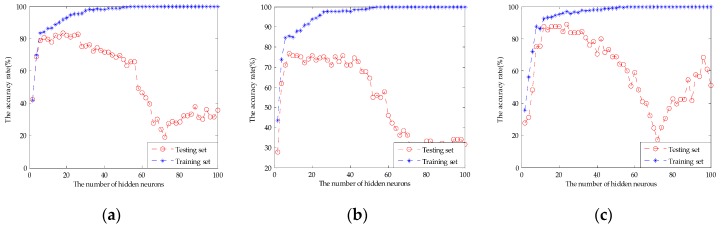
The classification performance of the ELM based on the number of hidden neurons: (**a**) e-tongue; (**b**) e-nose; and (**c**) the original fusion set.

Table 2 shows the classification results of single e-tongue, single e-nose, and the original fusion set based on the SVM, RF, and ELM. The classification accuracy rate of the SVM for e-tongue was 83.33%, for e-nose it was 80.56%, and the original fusion set was 88.89%. Figure 9 shows the parameter optimization process of the SVM based on GA. The best c and g were selected when the highest CVAccuracy was obtained to build the model. The classification accuracy rate of the RF for e-tongue was 83.33%, for e-nose it was 77.78%, and the original fusion set was 88.89%. Figure 10 shows the classification performance of the RF based on the number of decision trees. The classification accuracy rate of the ELM for e-tongue was 82.78%, for e-nose it was 78.89%, and the original fusion set was 88.33%. Figure 11 shows the classification performance of the ELM based on the number of hidden neurons. It can be seen that the classification accuracy rate increased by using data fusion. 

However, we cannot be sure that the 20-dimensional feature variables were the main variables. It is uncertain that each feature variable contributed to the beer overall flavor and affected the classification results. Therefore, the following three feature selection methods were discussed to find the main feature affecting the beer flavor.

**Figure 12:**
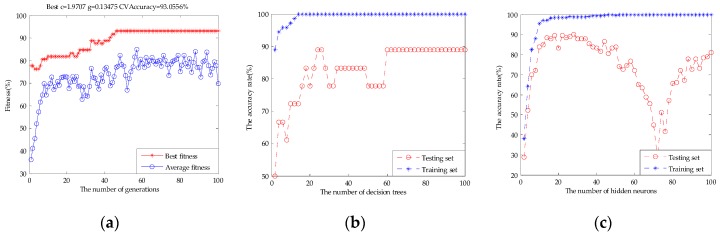
(**a**) The parameter optimization fitness curve of the SVM for the dimensionality reduction set by PCA; (**b**) the classification performance of the RF based on the number of decision trees for the dimensionality reduction set by PCA; and (**c**) the classification performance of the ELM based on the number of hidden neurons for the dimensionality reduction set by PCA.

**Figure 13:**
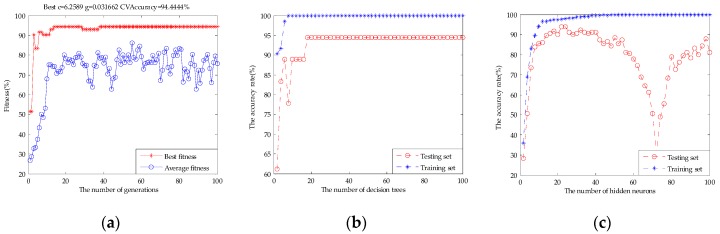
(**a**) The parameter optimization fitness curve of the SVM for the feature screening set by GA-PLS; (**b**) the classification performance of the RF based on the number of decision trees for the feature screening set by GA-PLS; and (**c**) the classification performance of the ELM based on the number of hidden neurons for the feature screening set by GA-PLS.

Table 3 shows the classification results of the original fusion set, the dimensionality reduction set by PCA, and the feature screening set by GA-PLS. It can be seen that the PCA extracted principal components did not work desirably to improve the classification results. SVM and ELM classification results rise slightly to 91.11% and 89.44%, respectively. The RF classification result was still 88.89%. This may be used as an unsupervised learning method without introducing classified information. It may lose effective authentication information and does not remove redundant information effectively. Figure 12 shows the parameter optimization fitness curve of the SVM, the classification performance of the RF based on the number of decision trees, and the classification performance of the ELM based on the number of hidden neurons for the dimensionality reduction set by PCA. However, compared with the 10 principal components extracted by PCA and the original fusion set, the 12-dimensional feature variables selected by GA-PLS obtained a better classification result. The classification accuracy rate of the SVM was 96.67%, for RF it was 94.44%, and for ELM it was 94.44%. This shows that GA-PLS removed some redundant information of the original fusion set and selected the effective feature variables. Figure 13 shows the parameter optimization fitness curve of the SVM, the classification performance of the RF based on the number of decision trees, and the classification performance of the ELM based on the number of hidden neurons for the feature screening set by GA-PLS. However, this method cannot find the combined behavior among variables, and cannot obtain the importance evaluation of each variables’ contribution.

**Figure 14:**
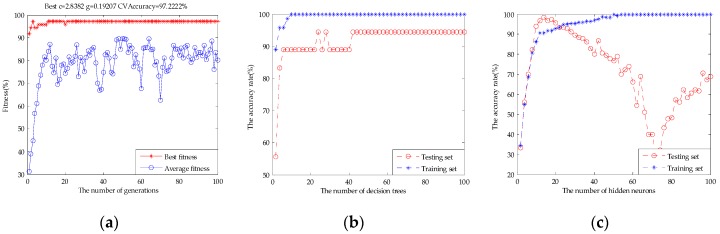
(**a**) The parameter optimization fitness curve of the SVM for subset #12; (**b**) the classification performance of the RF based on the number of decision trees for subset #11; and (**c**) the classification performance of the ELM based on the number of hidden neurons for subset #12.

Table 4 shows 20 subsets, which were generated with those variables based on the best VIP score. The classification performance of SVM and ELM in subset #7 and RF in subset #9 could be equal to the original fusion set, respectively, which meant that the original fusion set contained a large amount of redundant information. With the number of variables increased, the classification performance of SVM, RF, and ELM models appeared to have a small range fluctuation, and it showed that these variables had a relevant impact on the contribution of beer flavor features. The highest classification accuracy rate of SVM was up to 96.67% in subset #12, RF was 94.44% in subset #11, and ELM was 98.33% in subset #12, respectively. With the number of variables continuing to increase, the classification accuracy rate of the models decreased and did not exceed the highest value. Figure 14 shows the parameter optimization fitness curve of the SVM for subset #12, the classification performance of the RF based on the number of decision trees for subset #11, and the classification performance of the ELM based on the number of hidden neurons for subset #12. In this way, we not only obtained the best subset to achieve the purpose of reducing redundant variables, but also obtained the variables’ behavior by observing the classification tendency of the model when variables were added gradually and the highest classification accuracy rate for beer flavor information.

## 4. Conclusions

The main conclusions are as follows:

(1) Compared with the single e-tongue and single e-nose, the classification accuracy rate of beer flavor information was improved by using multi-sensor data fusion, and the classification accuracy rate of SVM was 88.89%, RF was 88.89%, and ELM was 88.33%;
(2) The feature selection method based on PCA did not obtain the best form of beer flavor information. The feature selection method based on GA-PLS improved the beer flavor classification rate and reduced the feature dimension obviously, and SVM showed the best classification performance at 96.67%. However, it did not give the contribution behavior of each variable for the overall information; and
(3) By variable accumulation based on the best VIP score, the classification accuracy rate of SVM and ELM in subset #7 was 88.89% and 88.33%, respectively, and the classification accuracy rate of the RF in subset #9 was 88.89%, which meant that the original fusion set contained a lot of redundant information. Finally, ELM showed the best classification performance 98.33% in subset #12. Thus, C00, AE1, W1C, W3S, W3C, W5C, W1W, CA0, cpa(C00), W2S, AAE, and W1S were considered as the main features.

Among the contributions of this study, a variable accumulation strategy based on the best VIP score was proposed and applied to beer flavor information identification. It provided a vital method, which used the least characteristic variables and the best fusion method, combined with excellent pattern recognition methods, to identify beer flavor information more efficiently and more accurately. It not only obtained the important evaluation of each variable, but also obtained the correlation behavior by observing the classification tendency of the model. Meanwhile, it also provided a more efficient and accurate method to monitor product quality in the actual process of industrialization.

## Figures and Tables

**Table 1:** Characteristics of sampled beers.

Brand	Alcohol Content (% vol)	Original Wort Concentration (° P)	Raw and Auxiliary Materials
Landai	≥4.3	11	Water, malt, rice, hops
Xuehua	≥3.3	9	Water, malt, rice, hops
Baiwei	≥3.6	9.7	Water, malt, wheat, hops
Harbin	≥3.6	9.1	Water, malt, rice, hops
Qingdao	≥4.3	11	Water, malt, rice, hops

**Table 2:** Comparison of results for single e-tongue, e-nose, and the original fusion set.

Dataset	Accuracy (%)		
	SVM	RF	ELM
E-tongue	83.33	83.33	82.78
E-nose	80.56	77.78	78.89
E-tongue and e-nose	88.89	88.89	88.33

**Table 3:** Comparison of results for different fusion feature sets.

Dataset	Accuracy (%)		
	SVM	RF	ELM
E-tongue and e-nose	88.89	88.89	88.33
PCA (e-tongue and e-nose)	91.11	88.89	89.44
GA-PLS (e-tongue and e-nose)	96.67	94.44	94.44

**Table 4:** Comparison of accuracy based on different classification models using different subsets of variables, based on VIP.

Subset	Variables	Accuracy (%)		
		SVM	RF	ELM
#1	C00	37.78	55.56	43.33
#2	C00 + AE1	71.67	66.67	78.89
#3	C00 + AE1 + W1C	76.11	66.67	73.89
#4	C00 + AE1 + W1C + W3S	74.44	77.78	80.56
#5	C00 + AE1 + W1C + W3S + W3C	77.22	72.22	78.89
#6	C00 + AE1 + W1C + W3S + W3C + W5C	76.67	77.78	80.56
#7	C00 + AE1 + W1C + W3S + W3C + W5C + W1W	88.89	83.33	88.33
#8	C00 + AE1 + W1C + W3S + W3C + W5C + W1W + CA0	88.33	83.33	86.11
#9	C00 + AE1 + W1C + W3S + W3C + W5C + W1W + CA0 + cpa(C00)	91.67	88.89	87.78
#10	C00 + AE1 + W1C + W3S + W3C + W5C + W1W + CA0 + cpa(C00) + W2S	82.78	83.33	86.11
#11	C00 + AE1 + W1C + W3S + W3C + W5C + W1W + CA0 + cpa(C00) + W2S + AAE	92.22	**94.44**	93.89
#12	C00 + AE1 + W1C + W3S + W3C + W5C + W1W + CA0 + cpa(C00) + W2S + AAE + W1S	**96.67**	94.44	**98.33**
#13	C00 + AE1 + W1C + W3S + W3C + W5C + W1W + CA0 + cpa(C00) + W2S + AAE + W1S + W2W	96.67	94.44	98.33
#14	C00 + AE1 + W1C + W3S + W3C + W5C + W1W + CA0 + cpa(C00) + W2S + AAE + W1S + W2W + W6S	96.67	94.44	93.89
#15	C00 + AE1 + W1C + W3S + W3C + W5C + W1W + CA0 + cpa(C00) + W2S + AAE + W1S + W2W + W6S + CT0	92.78	94.44	93.89
#16	C00 + AE1 + W1C + W3S + W3C + W5C + W1W + CA0 + cpa(C00) + W2S + AAE + W1S + W2W + W6S + CT0 + cpa(CT0)	91.67	88.89	92.78
#17	C00 + AE1 + W1C + W3S + W3C + W5C + W1W + CA0 + cpa(C00) + W2S + AAE + W1S + W2W + W6S + CT0 + cpa(CT0) + W5S	93.89	94.44	88.89
#18	C00 + AE1 + W1C + W3S + W3C + W5C + W1W + CA0 + cpa(C00) + W2S + AAE + W1S + W2W + W6S + CT0 + cpa(CT0) + W5S + cpa(AE1)	93.33	88.89	92.22
#19	C00 + AE1 + W1C + W3S + W3C + W5C + W1W + CA0 + cpa(C00) + W2S + AAE + W1S + W2W + W6S + CT0 + cpa(CT0) + W5S + cpa(AE1) + cpa(CA0)	87.78	88.89	87.78
#20	C00 + AE1 + W1C + W3S + W3C + W5C + W1W + CA0 + cpa(C00) + W2S + AAE + W1S + W2W + W6S + CT0 + cpa(CT0) + W5S + cpa(AE1) + cpa(CA0) + cpa(AAE)	88.89	88.89	88.33

Notes: the model with the best classification performance shown as bold data.

## References

[1] Denke M.A. (2000). Nutritional and health benefits of beer. Am. J. Med. Sci..

[2] Lynch K.M., Steffen E.J., Arendt E.K. (2016). Brewers’ spent grain: A review with an emphasis on food and health. J. Inst. Brew..

[3] Miranda C.L., Stevens J.F., Helmrich A., Henderson M.C., Rodriguez R.J., Yang Y.H., Deinzer M.L., Barnes D.W., Buhler D.R. (1999). Antiproliferative and cytotoxic effects of prenylated flavonoids from hops (Humulus lupulus) in human cancer cell lines. Food Chem. Toxicol..

[4] Pfeiffer A., Högl B., Kaess H. (1992). Effect of ethanol and commonly ingested alcoholic beverages on gastric emptying and gastrointestinal transit. J. Mol. Med..

[5] Stevens J.F., Ivancic M., Hsu V.L., Deinzer M.L. (1997). Prenylflavonoids from humulus lupulus. Phytochemistry.

[6] Murphy C., Cain W.S. (1980). Taste and olfaction: Independence vs interaction. Physiol. Behav..

[7] Murphy C., Cain W.S., Bartoshuk L.M. (1977). Mutual action of taste and olfaction. Sens. Process..

[8] Castro L.F., Ross C.F. (2015). Determination of flavour compounds in beer using stir-bar sorptive extraction and solid-phase microextraction. J. Inst. Brew..

[9] Dong J.J., Li Q.L., Yin H., Zhong C., Hao J.G., Yang P.F., Tian Y.H., Jia S.R. (2014). Predictive analysis of beer quality by correlating sensory evaluation with higher alcohol and ester production using multivariate statistics methods. Food Chem..

[10] Ghasemi-Varnamkhasti M., Mohtasebi S.S., Rodriguez-Mendez M.L., Lozano J., Razavi S.H., Ahmadi H., Apetrei C. (2012). Classification of non-alcoholic beer based on aftertaste sensory evaluation by chemometric tools. Expert Syst. Appl..

[11] Bacci L., Camilli F., Drago M.S., Magli M., Vagnoni E., Mauro A., Predieri S. (2012). Sensory evaluation and instrumental measurements to determine tactile properties of wool fabrics. Text. Res. J..

[12] Wang L., Niu Q., Hui Y., Jin H. (2015). Discrimination of rice with different pretreatment methods by using a voltammetric electronic tongue. Sensors.

[13] Ciosek P., Wesoły M., Zabadaj M. (2015). Towards flow-through/flow injection electronic tongue for the analysis of pharmaceuticals. Sens. Actuators B Chem..

[14] Cetó X., González-Calabuig A., Crespo N., Pérez S., Capdevila J., Puig-Pujol A., Valle M.D. (2016). Electronic tongues to assess wine sensory descriptors. Talanta.

[15] Romain A.C., Godefroid D., Kuske M., Nicolas J. (2005). Monitoring the exhaust air of a compost pile as a process variable with an e-nose. Sens. Actuators B Chem..

[16] Zhu J.C., Feng C., Wang L.Y., Niu Y.W., Xiao Z.B. (2017). Evaluation of the synergism among volatile compounds in oolong tea infusion by odour threshold with sensory analysis and e-nose. Food Chem..

[17] Li Q., Gu Y., Jia J. (2017). Classification of multiple chinese liquors by means of a QCM-based e-nose and MDS-SVM classifier. Sensors.

[18] Ghasemi-Varnamkhasti M., Mohtasebi S.S., Siadat M., Lozano J., Ahmadi H., Razavi S.H., Dicko A. (2011). Aging fingerprint characterization of beer using electronic nose. Sens. Actuators B Chem..

[19] Cetó X., Gutiérrez-Capitán M., Calvo D., Del V.M. (2013). Beer classification by means of a potentiometric electronic tongue. Food Chem..

[20] Banerjee R., Tudu B., Bandyopadhyay R., Bhattacharyya N. (2016). A review on combined odor and taste sensor systems. J. Food Eng..

[21] Men H., Chen D., Zhang X., Liu J., Ning K. (2014). Data fusion of electronic nose and electronic tongue for detection of mixed edible-oil. J. Sens..

[22] Zakaria A., Shakaff A.Y., Masnan M.J., Ahmad M.N., Adom A.H., Jaafar M.N., Ghani S.A., Abdullah A.H., Aziz A.H., Kamarudin L.M. (2010). A biomimetic sensor for the classification of honeys of different floral origin and the detection of adulteration. Sensors.

[23] Lu L., Deng S., Zhu Z., Tian S. (2015). Classification of rice by combining electronic tongue and nose. Food Anal. Methods.

[24] Han F., Huang X., Teye E., Gu F., Gu H. (2014). Nondestructive detection of fish freshness during its preservation by combining electronic nose and electronic tongue techniques in conjunction with chemometric analysis. Anal. Methods.

[25] Pan J., Duan Y., Jiang Y., Lv Y., Zhang H., Zhu Y., Zhang S. (2017). Evaluation of fuding white tea flavor using electronic nose and electronic tongue. Sci. Technol. Food Ind..

[26] Qiu S., Wang J., Gao L. (2015). Qualification and quantisation of processed strawberry juice based on electronic nose and tongue. LWT-Food Sci. Technol..

[27] Prieto N., Oliveri P., Leardi R., Gay M., Apetrei C., Rodriguez-Méndez M.L., Saja J.A. (2013). Application of a GA–PLS strategy for variable reduction of electronic tongue signals. Sens. Actuators B Chem..

[28] Zhi R., Zhao L., Zhang D. (2017). A framework for the multi-level fusion of electronic nose and electronic nongue for tea quality assessment. Sensors.

[29] Fu J., Huang C., Xing J., Zheng J. (2012). Pattern classification using an olfactory model with PCA feature selection in electronic noses: Study and application. Sensors.

[30] Hong X., Wang J., Qiu S. (2014). Authenticating cherry tomato juices—discussion of different data standardization and fusion approaches based on electronic nose and tongue. Food Res. Int..

[31] Cristiane M.D., Flavio M.S., Alexandra M., Antonio R.J., Maria H.O., Angelo L.G., Daniel S.C., Fernando V.P. (2017). Information visualization and feature selection methods applied to detect gliadin in Gluten-Containing foodstuff with a microfluidic electronic tongue. ACS Appl. Mater. Interfaces.

[32] Banerjee R., Tudu B., Shaw L., Jana A., Bhattacharyya N., Bandyopadhyay R. (2011). Instrumental testing of tea by combining the responses of electronic nose and tongue. J. Food Eng..

[33] Leardi R., González A.L. (1998). Genetic algorithms applied to feature selection in PLS regression: How and when to use them. Chemom. Intell. Lab. Syst..

[34] Fassihi A., Sabet R. (2008). QSAR study of p56(lck) protein tyrosine kinase inhibitory activity of flavonoid derivatives using MLR and GA-PLS. Int. J. Mol. Sci..

[35] Galindo-Prieto B., Eriksson L., Trygg J. (2015). Variable influence on projection (VIP) for OPLS models and its applicability in multivariate time series analysis. Chemom. Intell. Lab. Syst..

[36] Cortes C., Vapnik V. (1995). Support vector network. Mach. Learn..

[37] Qiu S., Wang J., Tang C., Du D. (2015). Comparison of ELM, RF, and SVM on e-nose and e-tongue to trace the quality status of mandarin (Citrus unshiu Marc.). J. Food Eng..

[38] Wei Z., Zhang W., Wang Y., Wang J. (2017). Monitoring the fermentation, post-ripeness and storage processes of set yogurt using voltammetric electronic tongue. J. Food Eng..

[39] Li Y., Zhang J., Li T., Liu H., Li J., Wang Y. (2017). Geographical traceability of wild boletus edulis based on data fusion of FT-MIR and ICP-AES coupled with data mining methods (SVM). Spectrochim. Acta Part A Mol. Biomol. Spectrosc..

[40] Breiman L. (2001). Random Forest. Mach. Learn..

[41] Nitze I., Barrett B., Cawkwell F. (2015). Temporal optimisation of image acquisition for land cover classification with random forest and MODIS time-series. Int. J. Appl. Earth Obs. Geoinf..

[42] Béjaoui B., Armi Z., Ottaviani E., Barelli E., Gargouri-Ellouz E., Chérif R., Turki S., Solidoro C., Aleya L. (2016). Random forest model and TRIX used in combination to assess and diagnose the trophic status of Bizerte Lagoon, southern Mediterranean. Ecol. Indic..

[43] Dubrava S., Mardekian J., Sadosky A., Bienen E.J., Parsons B., Hopps M., Markman J. (2017). Using random forest models to identify correlates of a diabetic peripheral neuropathy diagnosis from electronic health record data. Pain Med..

[44] Mao W., He L., Yan Y., Wang J. (2017). Online sequential prediction of bearings imbalanced fault diagnosis by extreme learning machine. Mech. Syst. Signal Process..

[45] Yu L., Dai W., Tang L. (2016). A novel decomposition ensemble model with extended extreme learning machine for crude oil price forecasting. Eng. Appl. Artif. Intell..

[46] Yang W.A., Zhou W., Liao W., Guo Y. (2015). Identification and quantification of concurrent control chart patterns using extreme-point symmetric mode decomposition and extreme learning machines. Neurocomputing.

[47] Maione C., Batista B.L., Campiglia A.D., Barbosa F.B., Barbosa R.M. (2016). Classification of geographic origin of rice by data mining and inductively coupled plasma mass spectrometry. Comput. Electron. Agric..

